# Comment on: Risk factors for hip displacement in cerebral palsy: A population-based study of 121 nonambulatory children

**DOI:** 10.1177/18632521231156548

**Published:** 2023-03-15

**Authors:** Philippe Wagner, Gunnar Hägglund

**Affiliations:** 1 Orthopedics, Department of Clinical Sciences, Lund University, Lund, Sweden; 2 Centre for Clinical Research, Uppsala University, Region Västmanland, Västerås, Sweden; 3 Department of Orthopedics, Skane University Hospital, Lund, Sweden

We read with great interest Drs Terjesen and Horn’s^1^ study on risk factors for hip displacement in cerebral palsy. This is an important topic, and we commend the authors for their work to move our knowledge on this subject forward.

In this prospective register study of 121 children in Gross Motor Function Classification System (GMFCS) levels III–V, <5 years of age at inclusion, the analysis showed initial migration percentage (MP) and MP velocity to be independently associated with hip displacement, defined as MP ≥ 40%. These associations were statistically significant (p ≤ 0.05). Meanwhile, associations with age, GMFCS level, head-shaft angle (HSA), and acetabular index were, in contrast to some earlier studies, not statistically significant (p > 0.05). The authors then concluded that the only variable that needs to be measured in hip surveillance, in order to predict the risk of hip displacement, is the MP.

We would like to draw attention to the fact that statistically non-significant results, in fact, are not evidence that an association between a studied variable and outcome does not exist. It only shows that there is a lack of empirical evidence in that particular study to support the existence of an association. The aphorism “Absence of evidence is not evidence of absence” summarizes this well-known fallacy, which has been described in the literature many times over the years. A famous example is by medical statisticians Douglas Altman and Martin Bland^2^ in their 1995 statistical notes in *BMJ* and perhaps most recently by Ranstam in an editorial in *Acta Orthopaedica* 2021.^3^

In the Terjesen and Horn study, the confidence intervals (CIs) of the non-significant associations of interest were quite wide. This implies that there is considerable statistical uncertainty with respect to the size of these associations. Consequently, the study provides little evidence that there are no associations, as this would require showing that the size of the odds ratios (ORs) were close to 1 with a narrow CI.

For example, examining the study results for cerebral palsy subtype (odds ratio (OR) = 2.98; 95% CI = 0.46–19.30), CIs indicate that the only reliable conclusion that can be reached is that one subtype group is associated with anywhere from roughly half the odds of hip displacement to 19.3 times higher odds compared to the other subtype group, in children with all other variables equal. Which group is estimated to be at higher odds is difficult to deduce as it is not clearly defined how variables are coded in the regression analyses. Furthermore, for initial HSA (OR = 1.01; 95% CI = 0.96–1.07), we can only conclude that the change in odds of hip displacement associated with a one degree increase in HSA is unlikely to be a decrease larger than 4% or an increase larger than 7%. This means that when comparing two groups of similar individuals differing by 20 degrees in HSA, the “high HSA group” could exhibit anywhere from a 56% decrease to a 3.9 times increase in odds of hip displacement. We do not believe that this is evidence that there is no difference between groups. Moreover, it would be difficult to argue that the wide range of values rule out any type of clinically relevant differences. The CI width of the remaining variables indicates the same type of uncertainty.

Therefore, we do not agree with the conclusion of this study. Although “initial MP” and “MP progression per year” may indeed be important risk factors of hip displacement, the study has not shown that they are the only ones out of the studied variables, and subsequently not that “The clinical significance of our results is that the only radiographic parameter that needs to be measured in routine hip surveillance in children below 5 years of age is the MP.” This question remains open.
